# Urease Inhibitors from *Nasturtium officinale* Extract for the Treatment of Urease Expressing Bacterial Infections

**DOI:** 10.1021/acsptsci.5c00809

**Published:** 2026-06-14

**Authors:** Rachel Heylen, Emily Owen, Kyle Stewart, Paul G. Winyard, Andrew Tobias A. Jenkins

**Affiliations:** † Department of Chemistry, 1555 University of Bath, Claverton Down, Bath BA2 7AY, U.K.; ‡ Watercress Research Ltd, Unit 24, Exeter Sky Park, Exeter EX5 2GE, U.K.

**Keywords:** *Nasturtium officinale*, urease, isothiocyanates, flavonoids, anti-inflammatory

## Abstract

Urease is a virulence factor associated with *Helicobacter pylori* infections, catheter-associated urinary tract infections, and incontinence associated dermatitis. Currently, few treatment options are available to treat urease-positive infections. Acetohydroxamic acid is the only licensed urease inhibitor, licensed in the USA under the name Lithostat. *Nasturtium officinale* (watercress) is a member of the *Brassicaceae* family. It has various potential medicinal properties which are little explored. In this study, its potential utility as a treatment option for urease-positive infections was tested. Various compounds present within *N. officinale* extract are potential urease inhibitors: In vitro, the ability of *N. officinale* extract to prevent accumulation of ammonia produced in *Proteus mirabilis* cultures was studied with two potential therapeutic mechanisms identified: 1. Inhibition of the urease enzyme, preventing the production of ammonia; 2. Sequestration of ammonia by isothiocyanates. Putative evidence of activity, in terms of protection of skin barrier function, was obtained in an in vivo human model, where the *N. officinale* extract was potentially able to prevent erythema caused by ammonia irritation. Finally in silico docking experiments were performed to investigate the binding of compounds found within *N. officinale* to *H. pylori* bacterial urease. The anti-inflammatory and antiureolytic properties of *N. officinale* extract could be utilized as a treatment against urease-positive infections.

## Graphical abstract

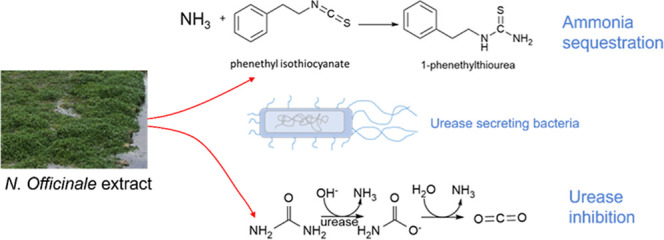

Urease is an enzyme expressed by various bacteria, fungi, and plants: it metabolizes urea to form two molecules of ammonia and carbonic aciid.^1^ The production of ammonia provides a nitrogen source for growth but also increases the pH of the surrounding environment.^1^ For bacteria, the increase in pH allows the establishment of a favorable environmental niche since many potential infection sites on the human body such as the skin and stomach are kept acidic as part of the innate immune system. This is particularly observed for *Helicobacter pylori*, a bacterium which infects the stomach that has a pH value of about 3.^2^ Urease also plays a role in urinary catheter blockage, whereby the increase in pH causes the precipitation of apatite and struvite crystals on the lumen and around the catheter, leading to blockage.^1^ The formation of the crystals allows the bacteria within the bladder to form extensive crystalline biofilms. These can form urinary stones. The infection can ascend the ureter causing pyelonephritis and in serious cases, urosepsis.^3^

A common urease-positive bacterium that is linked with catheter-associated urinary tract infections (CAUTI) is *Proteus mirabilis*. This bacterium is often found in the bladder of long-term catheterized patients. It’s ability to cause catheter blockage and the increase the risk of urosepsis, means it has an associated high mortality rate.^4^ Various other urease-positive bacteria, such as *Pseudomonas* spp., *Klebsiella* spp., *Staphylococcus* spp., *Citrobacter koseri*, and *Enterobacter* spp. are found in patients with CAUTI.^1,5^ Urease-positive microbes have also been associated with incontinence associated dermatitis (IAD) (nappy rash, diaper rash). Recently we described an IAD model whereby urease was hypothesized to be a pivotal virulence factor in the associated pathogenesis of IAD.^6^

Incontinence associated Dermatitis (IAD) has a complex pathogenesis.^6^ The proposed mechanism is that wet skin is exposed to enteric bacteria such as *P. mirabilis* which express the urease enzyme. Urease catalyzes the conversion of urea, found in urine and sweat to ammonia. Ammonia directly damages skin, in effect creating a chemical burn, possibly by damage to the tight cell junctions in the epithelium.^7^ The skin barrier is damaged and is more susceptible to frictional damage. The high pH environment from the ammonia promotes growth of other pathogenic microorganisms such as *Staphylococcus aureus* *,* increases the activity of proteolytic enzymes such as trypsin and induces a morphological change in commensal *Candida albicans*, allowing it to grow hyphae which it uses to cross the epithelium. Central to this model is urease: if it can be inhibited it should be possible to directly treat the cause of IAD, rather than its effect. The production of ammonia causes a significant rise in skin pH, which can lead to an unfavorable dysbiosis of the skin microbiome and activation of fecal enzymes, such as trypsin, leading to epidermal degradation.^8–10^ Furthermore, ammonia can trigger epidermal keratinocytes to release growth factors and pro-inflammatory cytokines, including IL-1α, IL-8 and tumor necrosis factor-α (TNF-α), all of which are involved in the damage during the observed inflammatory response linked to IAD.^8,11^

Various urease inhibitors have been identified, including *N*-(*n*-butyl) thiophosphoric triamide (NBPT), which has been added to nitrogen fertilizers to prevent nitrogen loss.^12^ Acetohydroxamic acid (AHA) is the only licensed urease inhibitor for human use, licensed in the USA, Kuwait, and Spain under the name Lithostat.^13–15^ It has been prescribed to treat catheter encrustations, as well as hyperammonemia caused by *H. pylori* infections.^16^ Despite its license, AHA is rarely used in the clinic owing to its toxic side effects: hemolytic anemia and teratogenesis.^15,17^

Natural products have also been identified as urease inhibitors including flavonoids. For example, quercetin has a reported IC_50_ of 11.2 μM against *H. pylori* urease.^18^ The therapeutic use of a *Nasturtium officinale* extract is advantageous because it contains multiple active compounds which may have urease inhibitory properties. *N. officinale*, watercress, has been identified as a medicinal plant with historical use in traditional medicine in Iran, Azerbaijan, Morocco, and Mauritius.^19^ It has been investigated for its pharmaceutical use and has demonstrated: antioxidant, anticancer, antibacterial, anti-inflammatory, and cardioprotective properties. The chemical composition of *N. officinale* stem and leaf extracts includes: polyphenols,^20,21^ saponins, isothiocyanates,^22^ glucosinolates,^23^ palmitic acid, monoterpenoids, sesquiterpenoids, and various vitamins and their derivatives^19,24^

Potentially, urease can be inhibited through multiple mechanisms, including both competitive and noncompetitive binding. Plant flavonoids have emerged as promising natural urease inhibitors, with multiple studies demonstrating their ability to suppress urease activity through interactions with the enzyme’s nickel-containing active site. Recent reviews highlight that various flavonoid structures, including quercetin, apigenin-, chrysin- and luteolin-based glycosides, exhibit significant antiurease effects and may be optimized through structural modifications to enhance potency.^25^ Studies of *Passiflora edulis* further confirm that flavone *C*-glycosides can inhibit urease by up to 57%, suggesting their relevance in both agricultural and biomedical applications.^26^

Flavonoids have low solubilities in water, however within plant extract containing fatty acids such as palmitic acid their solubilities are improved.^27,28^ Human trials of *N. officinale* extract did not report any adverse side effects or toxicity.^24,29^ The recent review by Kimek-Szczykutowicz et al. of *N. officinale*, stated that it had undiscovered biological properties, that could potentially be utilized in novel therapeutic approaches.^19^ A study by Sotoris et al. quantified the composition of *N. officinale* extract finding gluconasturtiin, phenethyl isothiocyanate, quercetin-3-O-rutinoside and isorhamnetin.^30^

The work detailed in this paper aimed to study the interaction of putative urease inhibitory molecules found in watercress extract by quantifying the inhibitory effect of watercress extract on urease catalyzed ammonia production; measure the ability of watercress extract to sequestrate ammonia and establish whether watercress extract is able to reduce pH and erythema caused by *P. mirabilis* inoculation in vivo on human volunteer skin; studying their interaction with the urease active site *in-silico*. The potential antiureolytic and ammonia sequestrating properties of *N. officinale* extract are studied in relation to the potential use of the extract as a treatment against urease-expressing bacterial infections, specifically those associated with CAUTI and IAD.

## Results and Discussion

### Urease Inhibition by Watercress Extract

*P. mirabilis* is a well-studied urease-positive microorganism. We therefore tested the *N. officinale* extract against whole-cell cultures of *P. mirabilis* to assess the extract’s ability to inhibit urease activity. Urease is intracellular in *P. mirabilis*. The current assay also tests the ability of *N. officinale* extract to cross the membrane of the bacteria and access the urease enzyme.^31^ Previous work has suggested that quercetin is unable to cross the membrane in *P. mirabilis*. Within the *N. officinale* extract, the presence of fatty acids such as palmitic acid may improve the solubility of the flavonoids and thus allow transport through the membrane. Results from the inhibitory assay demonstrated that <10% (v/v) of extract was sufficient to inhibit *P. mirabilis* urease (Figure 1). *N. officinale* extract is not a known antimicrobial, therefore it is unlikely to present resistance pressures on bacteria (Supporting Information Figure S5). This provides a potential therapeutic advantage, as it appears possible that the extract does not damage commensal flora, but still provides inhibition of the virulence factor, urease, thereby preventing associated pathologies. Note residual activity refers to the activity of urease following addition of watercress extract, normalized to 100% activity at zero watercress concentration. Note the very low measurement of urease activity at 2% watercress concentration was a reproducible (*n* = 3) anomaly which is unexplained.

**Figure 1:**
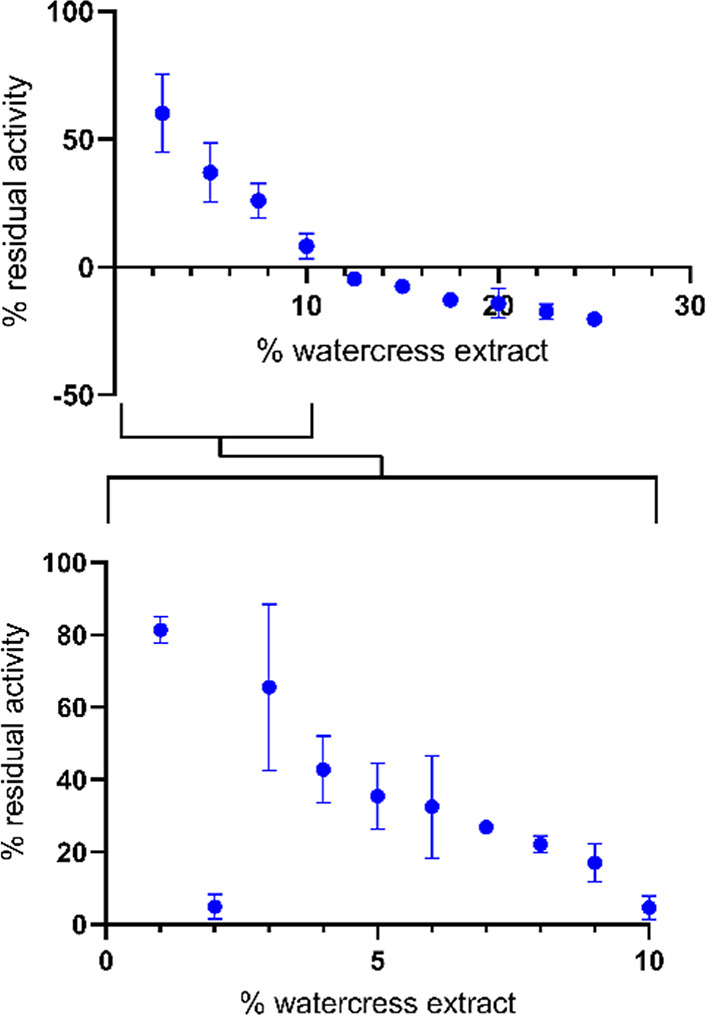
Assay of the urease activity of *Proteus mirabilis* as a function of watercress extract concentration, showing the effectiveness of the extract as an inhibitor of the bacterial urease. Experiments were completed with three biological repeats, with each experiment consisting of two technical repeats. The graphs show the mean values of the biological repeats, with error bars representing the standard deviations.

### Ammonia Scavenging

The urease inhibition assay showed that *N. officinale* extract with a concentration greater than 12% (v/v) demonstrated a negative measurement of % residual activity (Figure 1). This may suggest that an additional mechanism is involved: it was hypothesized that the *N. officinale* extract was able to scavenge the existing ammonia. A chemical reaction between isothiocyanates and ammonia is well established.^32^ To measure the potential ammonia scavenging by the *N. officinale* extract, the urease activity assay (which measures accumulation of ammonia) was used without the presence of urease to determine ammonia sequestration properties. The concentration of residual ammonia decreased when increasing concentrations of *N. officinale* extract were added (Figure 2). Therefore, it appears the extract directly scavenges ammonia in addition or in place of inhibiting urease. A concentration of 20% (v/v) *N. officinale* extract was sufficient to sequester the excess ammonia. It appears that both urease inhibition and ammonia sequestration play a role in the ammonia-lowering activity of *N. officinale* extract. The ammonia scavenging may be advantageous as the extract may potentially sequester the existing ammonia formed by an established infection. Ammonia is pivotal in causing the increase in pH which leads to urinary catheter blockage in patients with long-term indwelling catheters (Stickler and Feneley, 2010) as well as directly damaging skin.^33^ Ammonia is also essential in allowing *H. pylori* to establish its infection within the stomach where it protects the bacterium by buffering the stomach acid.^34^ Therefore, this sequestration property of the *N. officinale* extract suggests the possibility of treating urease-positive infections via more than one mechanism. The apparently negative corrected ammonia concentration at >20% watercress is less than two standard deviations lower than zero and suggests the measured ammonia concentration at 25% watercress extract or higher is zero.

**Figure 2:**
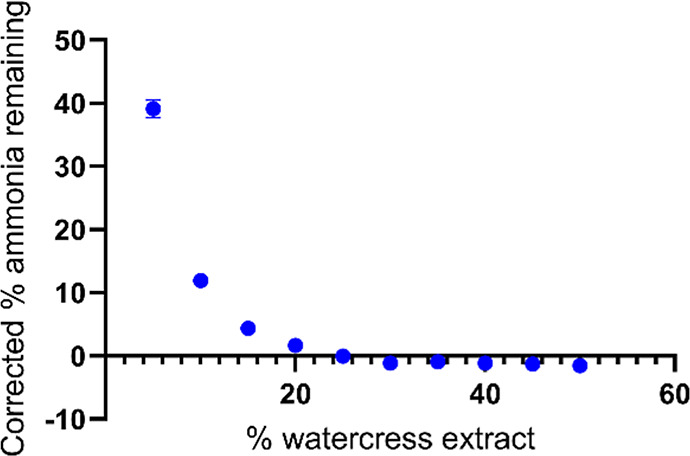
Ammonia scavenging effect of the watercress extract. The graph shows the percentage of ammonia present after incubation of a solution of ammonium chloride with varying concentrations of *Nasturtium officinale* extract. Experiments were performed with three biological repeats, with each experiment consisting of two technical repeats. The graph shows the mean values of the repeats with error bars representing the standard deviations. Where error bars are not visible, this is because the data point is larger than the size of the error.

We hypothesized that ITCs react with ammonia to form a thiourea, thereby sequestering ammonia (Figure 3). To test this, synthetic phenethyl isothiocyanate (PEITC) was mixed with aqueous ammonium chloride. The NMR spectrum of PEITC was compared to that of the expected product, 1-phenethylthiourea, demonstrating the formation of the thioamide bond (Supporting Information Figure S6). PEITC was found to be a principal component of watercress extract. Therefore, it is likely that the formation of the thioamide bond via this mechanism accounts for the sequestration of ammonia.^24^

**Figure 3:**
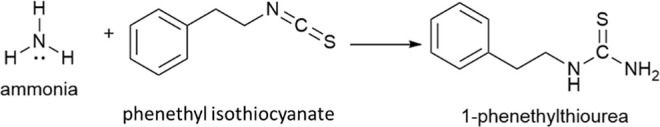
Mechanism of ammonia sequestration by isothiocyanates. Phenethyl isothiocyanate reacts with ammonia to form 1-phenethylthiourea.

### Impedance Measurement of Ex-Vivo Porcine Skin Barrier Function Change

Ammonia has been shown previously to directly damage skin barrier functions and this process has been central to the pathogenesis of IAD.^35^ The utility of impedance spectroscopy for measuring the breakdown in skin barrier function when exposed to urease and artificial urine has been detailed in a previous publication (Figure 4 shows the measured skin resistance divided by the baseline skin resistance at the start point of the experiment for three time points (*R*_Time_/*R*_Baseline_). A small decrease in the resistance of skin exposed to artificial urine is seen, as would be expected due to hydration of the stratum corneum. Skin inoculated with *P. mirabilis* alone showed a rapid decrease in resistance with a greater than 95% decrease by 6 h. Skin pretreated with 20% watercress extract showed a very different change: at 2 h measured resistance appeared statistically unchanged from the baseline (unlike *P. mirabilis* inoculated skin with a 70% decrease); by 4 h the apparent protective effect of watercress extract had declined, but still provided a statistically significant improvement in resistance (*p* < 0.05) compared with the *P. mirabilis* inoculated skin; at 6 h, there was still a protective effect, although the effect was no longer statistically significant.

**Figure 4:**
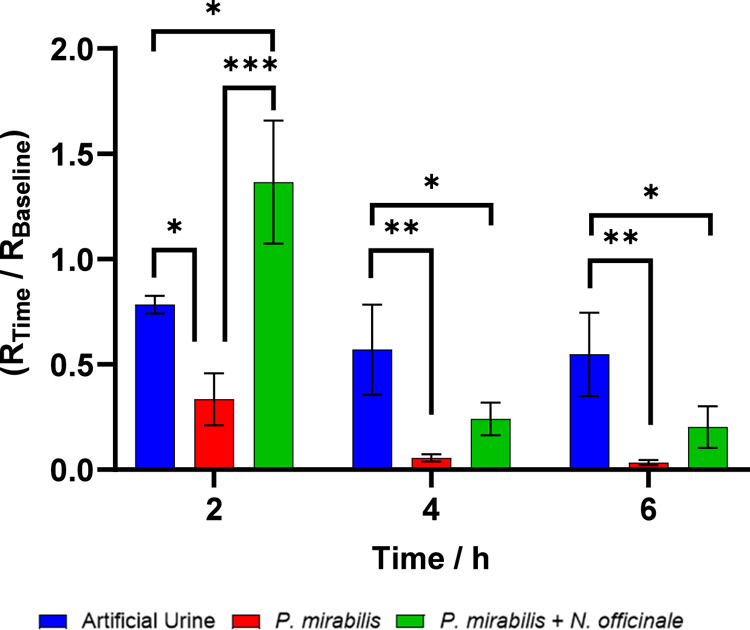
Stratum corneum resistance of ex vivo porcine skin, subjected for 2–6 h to artificial urine with/without *Proteus mirabilis* B4 was tested with/without 20% *Nasturtium officinale* extract. Fitted resistance ‘*R*_2_’, from an *R*_1_(*R*_2_Q) circuit model, was normalized (*R*_Time_/*R*_Baseline_). Error bars represent the standard deviation of three independent replicates, analyzed on GraphPad Prism 10 using an Ordinary One-Way ANOVA: *p* ≤ 0.05 (*), *p* ≤ 0.01 (**) and *p* ≤ 0.001 (***).

### In-Vivo Impedance Measurement of Efficacy of Watercress Extract on Human Skin

Previous work has shown that the in vivo human ventral forearm is a safe and effective model for IAD. Human skin has the advantage that, being vascularized and with a functioning immune response, it also responds with visible erythema.^35^ In this study 1 M ammonia solution in artificial urine was applied to the skin for 2 h and the change in skin barrier function measured using impedance spectroscopy. Figure 5 shows the normalized results, together with a photograph of the erythema produced by contact with ammonia. Further details in Supporting Information Figure S7.

**Figure 5:**
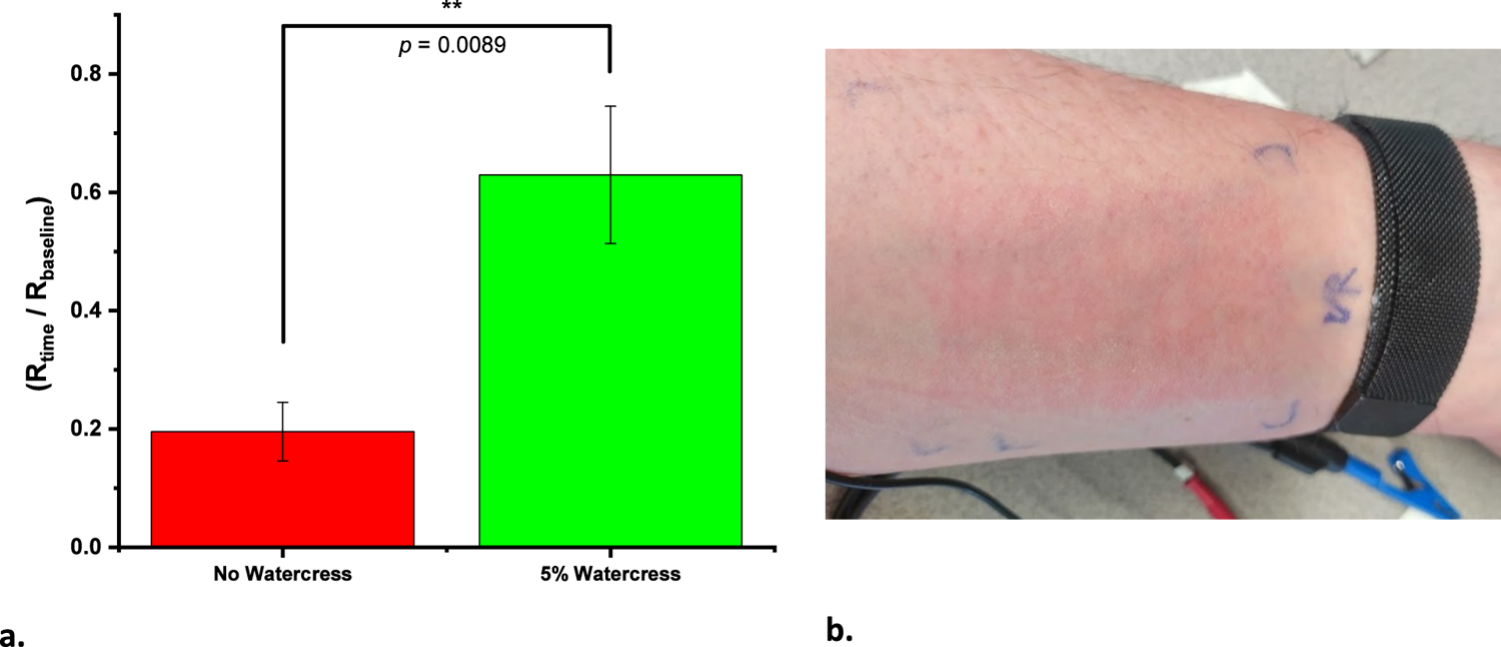
(a). Ratio of skin baseline resistance and resistance after 2 h exposure to 1 M ammonia solution (pH 9.2) showing the potentially protective effect of watercress extract. Analyzed with a *t*-test on Graphpad Prism, four replicates on different skin sites. (b). Erythema on ventral forearm following exposure of skin patch to 1 M ammonia.

### In silico Docking Data

In order to better understand potential inhibitory mechanisms of small molecule components of watercress extract *in-silico* modeling was used to look at the docking of putative inhibitory molecules with cysteine residues close to the active site of urease, in this case bacterial urease from *H. pylori* which has its crystal structure published (Protein Data Bank (PDB) = 1E9Y).

In silico docking methods allow ligands to be computationally docked on to the surface of the crystal structure. Examination of close binding contacts allows predictions to be made about methods of inhibition. Ligands expected to be present in aqueous *N. officinale* extract were docked onto the urease. A sequence alignment allowed a comparison of different ureases (Supporting Information Figure S1). There is more variation in the amino acids involved in the active site flap. Different flexibility is observed depending on the presence of proline residues. However, generally, the active site flap is well conserved between bacterial species (Supporting Information Figure S1). Due to this similarity, the results from these docking experiments can be compared to other bacterial ureases.^3,36^

Identifying all the compounds within aqueous *N. officinale* extract is difficult due to inherent variability in extract preparation and growing conditions.^22^ Therefore a list of compounds to dock to the urease was generated by analysis of the literature (Supporting Information Table S1). Lead Finder (LF) dG is a docking score which predicts the energy involved in the ligand–protein binding interaction, the more negative the score the stronger the binding. Larger molecules generate a larger docking score even though they might not necessarily exhibit stronger binding.^37^ Therefore, to assess the ligand potential, both the docking score and the position of binding was taken into account. Initially, AHA and urea were docked into the active site of urease to assess the accuracy of the docking experiments. The docking occurred as expected (Supporting Information Figure S2).

Some of the isothiocyanate (ITC) constituents of *N. officinale* have known urease inhibitory properties.^19,24,38^ It was predicted that these compounds could form covalent bonds with cysteine residues in the urease by direct reaction of the -N = C—S group with cysteine to form a thiourea covalent adduct. Covalent inhibitors are rarely seen in the clinic owing to their associated toxicity. However, they can be effective, with high potency.^39^ Butynedioic acid was identified as a covalent inhibitor, predicted to bind to the Cys-322 residue from *Sporoscarcina pasteurii* urease.^40^

The cysteine residues found on the surface of *H. pylori* urease were identified as potential therapeutic targets, as these would be more accessible to ITCs (Supporting Information Figure S3). Three series of ITCs (i.e., methylthio-, methylsulfinyl- and benzyl-ITCs), predicted to be present in the *N. officinale* extract, were prepared and docked to each cysteine residue identified on the surface of the urease: Cys-153, Cys-257, and Cys-3201 (Supporting Information Table S1, Supporting Information Figure S3). The LF dG score for each of the ITCs was compared (Figure 6). Docking onto Cys-153 and Cys-321 generally appeared to be more favorable than Cys-257 (Figure 6). The docking score was comparable between each of the three series, a general increase in the docking score was observed as the length of the ITC was increased, up to 7 carbons (heptyl). However, this pattern was not always observed, as in the binding of the methylsulfinyl-ITC series to Cys-257 (Figure 6, panel B).

**Figure 6:**
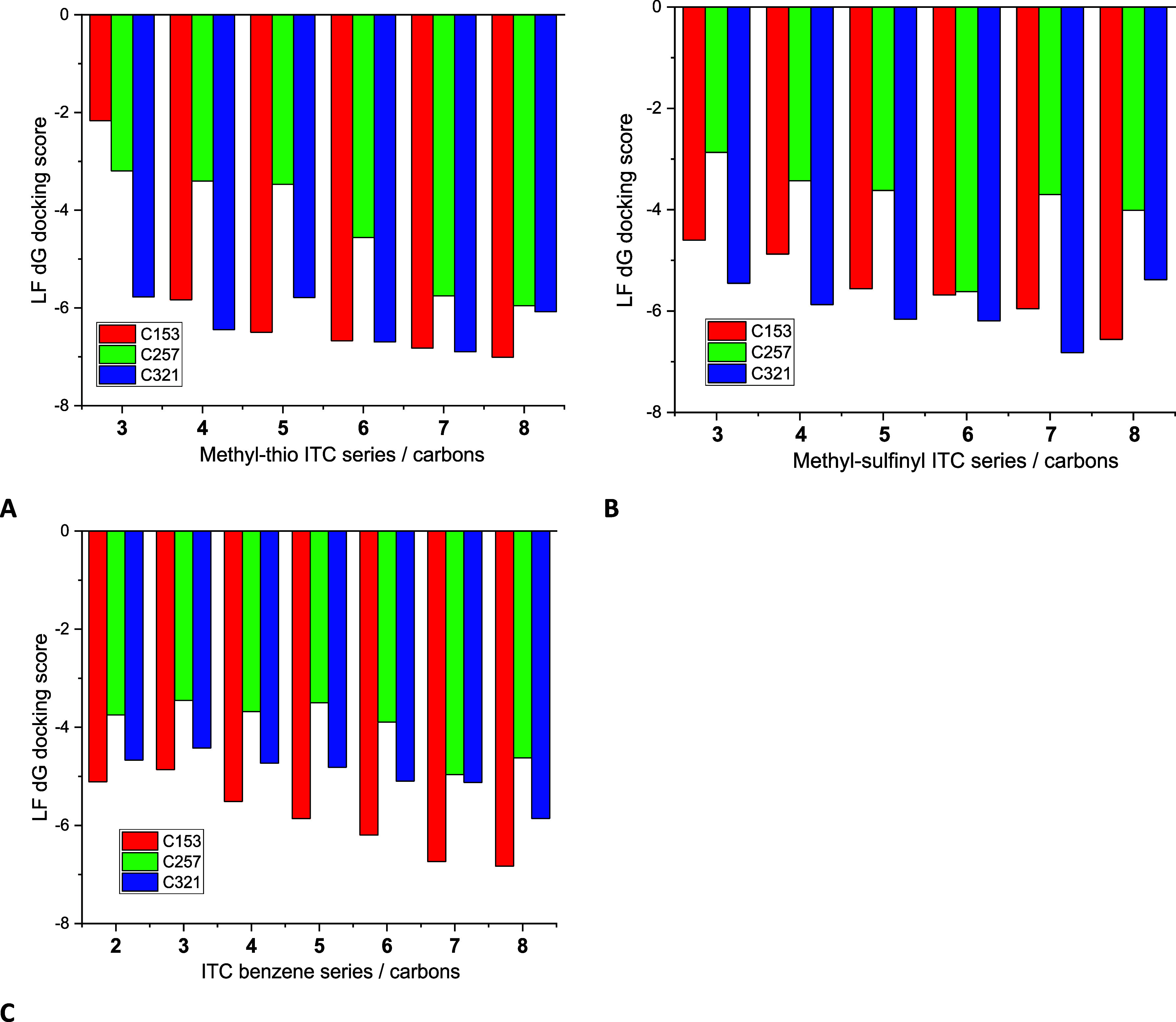
Comparison of the docking scores for covalently docked isothiocyanates. (A). Methylthio-ITC series. (B). Methylsulfinyl-ITC series. (C). Benzyl-ITC series. *X*-axis numbers correspond to the number of carbons in the ITC chains, and docking scores are recorded in Supporting Information Table S3. The compounds were computationally docked on to urease from *Helicobacter pylori* using Cresset Flare v. 4.0.2 software.

Docking onto Cys-321 was the most interesting as this cysteine residue is part of the active site flap. The ligands docked here appeared to bind in toward the active site. An in vitro analysis of ITC compounds: 4-(methylsulfinyl)­butyl-NCS reduced the activity of *H. pylori* urease to 36% after 30 min of incubation.^38^ In the present study, a LF dG score of −5.874 was measured for this compound (Figure 6, panel B).^38^ However, an in vitro analysis of 8-(methylsulfinyl)­octyl-NCS, 5-(methylsulfinyl)­phenyl-NCS, and 2-phenylethyl-NCS, Fahey et al. did not measure inhibitory activity against urease, despite comparable docking scores measured in the in silico analysis.^38^ This indicates the importance of in vitro analysis alongside in silico docking experimentation and suggests that ITCs may have a mode of action which is not due to urease inhibition (or not solely due to) but rather due to ammonia sequestration (see Section 2.2). ITCs are produced in the plant extract by mechanical disruption of the cells and activity from the myrosinase enzyme, which metabolizes glucosinolates.^41^ Glucosinolates are found throughout the plant: stems, leaves, roots, and also in the flowers; consequently *N. officinale* could be harvested and processed at different times and using different mechanical disruption techniques which could optimize the production of ITC compounds.^19^

Flavonoids are another group of compounds present in *N. officinale* extracts.^20^ Previous work (Xiao et al., 2012) predicted that flavonoids would associate with Cys-321 in the active site flap.^42^ Various flavonoids were docked into a grid prepared around Cys-321. As flavonoids are larger molecules compared to the relevant ITCs, they tend to have a higher docking score since they make more contacts with the protein, but this does not necessarily mean they are better inhibitors.^37^ The flavonoids gave higher docking scores compared to the ITC ligands (Figure 7, panel A). Quercetin 3-sophoroside (QOS) had the highest docking score and it appears to bind near the active site of the urease, while making contacts with the active site flap (Figure 7, panel B). Quercetin has already been identified as a urease inhibitor and computational docking completed by Xiao et al.^42^ suggested that quercetin interacted with the active site flap. However, the docking experiment here suggested that the phenyl moiety did make contact within the active site, having a weaker interaction with the active site flap (Supporting Information Figure S4). This indicates the variability in the in silico docking software and emphasizes the importance of backing up the in silico data with in vitro experimentation.

**Figure 7:**
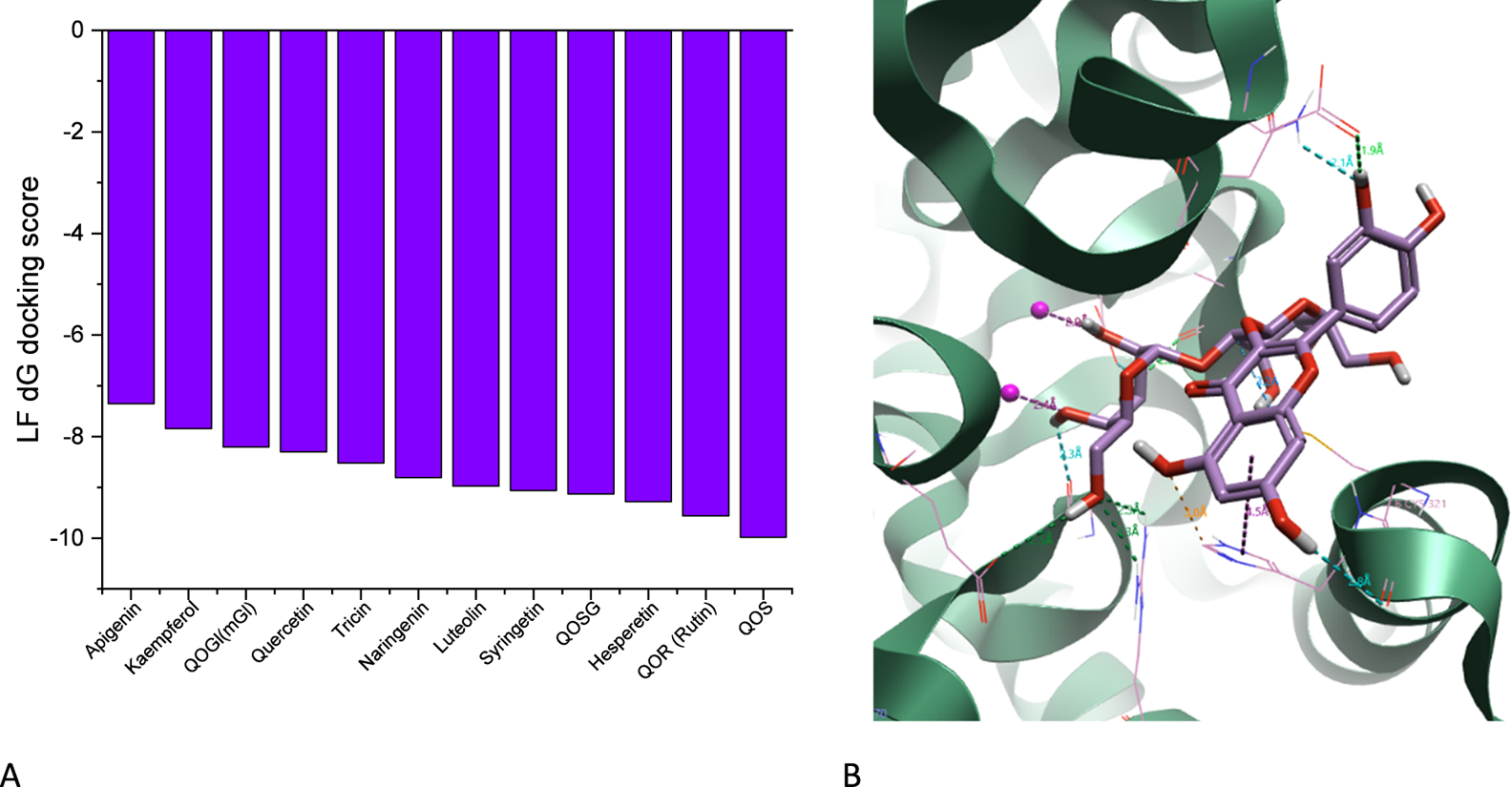
In silico flavonoid docking to *Helicobacter pylori* urease. (A). Comparison of LF dG docking scores of flavonoid derivatives against urease. (B). Quercetin 3-sophoroside docked to urease. The contacts formed between the Ni^2+^ ions, and amino acid residues are Asp-165, Asn-168, Glu-222, Gly-279, Cys-321, His-322, Arg-338, Asp-362, and Ala-365. Close contact amino acids shown in thin line, docked ligand as thick lines. The molecules were docked using Cresset Flare v. 4.0.2 software. The images were generated using Flare from Cresset. Abbreviations: QOS = Quercetin-3-O-sophoroside; QOSG = Quercetin-3-O-sophoroside, 7-O-glucoside; QOGl­(mGl) = Quercetin-3-O-Glc-(6′-malonyl-Glc); QOR: Quercetin-3-O-rutinoside (Rutin).

The acute toxicity of ammonia is well-known, but its role in various pathologies is perhaps less well appreciated. Ammonia is associated with skin damage in IAD, to damage to the stomach lining, and putative role in inflammatory bowel disease. Ammonia is primarily made by the action of bacterial urease enzyme converting urea but can also be a biproduct of certain liver diseases. Given the multiple roles of ammonia in human disease it is perhaps surprising that there are so few drugs which target it: only acetohydroxamic acid is currently licensed for clinical use, and only in some countries due to its toxicity.^43^ There is interest in developing new urease drugs, with a range of strategies being explored, one issue however will be the water solubility of various of the proposed compounds.^44^ For example, the phenylethyl ITCs explored in this paper are themselves of low water solubility, which would necessitate emulsification or using other strategies to ensure they reach their target if used as single component drugs. Moreover, the high reactivity of many synthetic ITCs with amines limits their utility as drugs.

Using naturally derived sources of urease inhibitors and ammonia sequestering molecules allows for the possibility of having a matrix of plant derived fatty acids and lipids which help to solubilize and stabilize active moieties such as PEITC found in the plant extract. *N. officinale* extract is a complex mixture of various chemicals including lipids, fatty acids and crucially both isothiocyanates and flavonoids. It is likely that there are important synergies between these compounds, which allows for both solubilization of relatively insoluble components, such as flavonoids and ITCs by lipids and fatty acids, but *in-silico* modeling and chemical analysis suggests putative mechanisms of action for various quercetins as urease inhibitors and ITCs as ammonia scavengers.

## Materials and Methods

### Plant Material, Extraction and Analysis

Watercress used in this trial was obtained from The Watercress Company (Dorchester, Dorset DT2 8QY). The supplied plant material (10 kg of dark green variety) was harvested to a specification of 20 cm length. Watercress was refrigerated in a cold room at 4 °C for 24 h before the extraction process began. The watercress was prepared using a RoboQbo 15-4 food processor, which has a sample bowl equipped with a cutting blade. Using a cutting blade, speed and residence time were investigated to determine the optimum conditions to homogenize the material. The bowl was loaded with 0.5 or 1.0 kg of watercress and chopped for 5 min at 2000 rpm. Using these cutting conditions, samples of the homogenized mixture were taken at time intervals of 0 min (immediately after cutting), 5 min, 10 min, 30 min, 1 h, 2 h, 3 h, 6 and 7 h. Samples were collected and sealed in a 50 mL sample tube then immersed in a water bath at 80 °C for 1 h. Samples were then centrifuged at 14,000 *x g* for 10 min and filtered through a 0.25 μm PTFE syringe filter. Filtrate was collected and 1 mL aliquots transferred to Eppendorf tubes and stored at −20 °C before analysis.^45^

### In silico Docking Experimentation

The crystal structure of *H. pylori* urease (PDB: 1E9Y) was used to complete the docking experiments, it had a high resolution of 2.8 Å, providing a sufficient crystal structure for docking experiments (N. Ha et al., 2001). The crystal structure was prepared and docking experiments were completed using Cresset^©^ Flare 4.0.2 (Revision: 40719, Cresset, Litlington, Cambridgeshire, UK). Ligands were prepared using ChemDraw 19.1.1.21 (PerkinElmer Informatics, Waltham, Massachusetts, US), and docked using the ‘accurate but slow’ setting.^46^ A grid box for docking ligands was designed with a 10 Å cube around the active site, for ligands designed to target the active site, this was altered depending on the predicted binding site. AHA, a ligand that has been crystallized with urease, was computationally docked and compared to the crystallized structure, to check the docking parameters.^36^ The flavonoid grid box included the active site around C321. The isothiocyanates were covalently docked to cysteine residues identified on the surface of urease: C153, C257, and C321. Ligands were visually examined for their close interactions with the urease and the LF dG was used to compare the ligands, LF dG which has been optimized for protein–ligand binding energy (on the assumption it has the correct pose), the more negative the LF dG the stronger the predicted binding.

### In vitro Urease Activity Assay

Urease activity was measured by determining the ammonia accumulation using the Berthelot assay.^47^ *N. officinale* extract was tested against *P. mirabilis* B4 (Jenkins Group collection, University of Bath). *P. mirabilis* was cultured overnight in Luria–Bertani (LB, Merck, Germany) at 37 °C with 200 rpm shaking. The overnight culture was centrifuged at 3100 *g*, for 10 min at 4 °C and the supernatant discarded, and the pellet resuspended in PBS (Fisher, Scientific, UK). *N. officinale* extract was stored at −20 °C and defrosted at room temperature. Difference dilutions of *N. officinale* extract were prepared in phosphate buffer (100 mM sodium phosphate, pH 7.4). Different dilutions of *N. officinale* extract (100 μL) were incubated with *P. mirabilis* culture (100 μL) and urea (200 μL, 50 mM urea in phosphate buffer) for 30 min at 37 °C. This was completed using three independent biological repeats. In a 96-well plate (Corning, UK), 0.5% (v/v) sulfuric acid (10 μL) was added to each well. After incubation, the solution was added to the 96-well plate (20 μL, 2*x* technical repeats), 60 mM sodium hydroxide (20 μL) was added to each well. Solution A (50 μL 106 mM phenol, 191 μM sodium nitroprusside) and solution B (50 μL, 125 mM sodium hydroxide, 125 mM sodium hypochlorite) was added to each well. The plate was incubated in foil for 30 min, at 37 °C. The absorbance was read at 636 nm (SPECTROstar Omega BMG LabTech, Germany. Urease activity was calculated as follows: Urease activity % = (*A*_test_–*A*_neg_)/(*A*_postive_–*A*_neg_), where negative control was absorbance without *N. officinale* extract and without *P. mirabilis*; positive control is with *P. mirablis* but without *N. officinale* extract.

### Minimum Inhibitory Concentration

*P. mirabilis* cultures were grown as described previously in Section 4. *N. officinale* extract was diluted in LB media. To the first column on a 96-well plate (Corning, UK), 200 μL of *N. officinale* extract was added. It was then serially diluted 2-fold (100 μL) across the plate to column 10. A subculture of *P. mirabilis* (1 × 10^6^ CFU mL^–1^), 100 μL, was added to the first 10 columns. LB broth only (100 μL) was added to column 11, which constituted the negative control. *P. mirabilis* subculture (200 μL) was added to column 12, which was the positive control. The plate was incubated at 37 °C for 18 h. Using a SPECTROstar Omega plate reader (BMG LabTech), the absorbance at 600 nm was measured at regular time points during the 18 h incubation. The plate was shaken for 10 s, at 200 rpm prior to a reading being taken. No inhibition of bacterial growth by watercress extract was measured over the concentration range 0.2–50%.

### Ammonia Scavenging

To test for ammonia scavenging, the same Berthelot assay was used as described in Section 4. However, instead of adding *P. mirabilis* to provide a urease source, ammonium chloride was added (7 mM, in phosphate buffer, 100 mM sodium phosphate, pH 7.4). The reaction of PE-ITC (3.06 mM, Merck, Germany) with excess ammonia hydroxide (30.6 mM, 35% (v/v), Fisher Scientific) was performed for 72 h with stirring in methanol (reaction volume 10 mL). PE-ITC and the resulting product were analyzed by ^1^H NMR spectroscopy using a Bruker 500 MHz spectrometer in CD_3_OD solvent. Resulting spectra were analyzed and processed using TopSpin 4.0.8.

### Ex-Vivo Porcine Skin Impedance Measurements

Details of the impedance protocol plus electrodes and procedure are provided in a previous publication (Owen et al., 2023). Briefly, porcine skin was shaved and inoculated as detailed in the in vivo study, above. 3 M Red Dot ECG electrodes were placed on the skin and impedance measured using a PalmSens4 potentiostat (PalmSens, The Netherlands) between 50 kHz to 1 Hz, with spectra being fitted to an *R*_1_(*R*_2_Q) equivalent circuits using the PSTrace 5.8 software, where the magnitude of *R*_2_ relates to the stratum corneum resistance. Impedance spectra of the skin was measured at 0, 2, 4, and 6 h with the normalized change in *R*_2_ being recorded as *R*_baseline_ at *t* = 0 and *R*_Time_ for the three time points, with *R*_Time_/*R*_baseline_ being plotted as a function of skin treatment condition and time.

### In-Vivo Skin Impedance Measurement of Watercress Extract

Experiments using an in vivo model urine and ammonia driven skin inflammation were approved by the Research Ethics Committee for Health at the University of Bath (EP 22 0006). Artificial urine was prepared according to Nzakizwanayo et al.^48^ Watercress extract was diluted to 10% in deionized water and applied to human volunteer ventral forearms using cotton wool. The skin was allowed to dry and then exposed to 1 M ammonia solution in artificial urine (pH 9.2) by soaking a 3 × 2 cm patch of Aquacell hydrogel dressing (Convatec Ltd.) and affixed to skin using an occlusive film (Mölnlycke Mepore film) for 2 h.^6,35^ After 2 h the skin was allowed to dry for 20 min and skin impedance measured using ECG electrodes fitted to an *R*_1_(*R*_2_Q) equivalent circuit model and *R*_2_ taken as the resistance primarily related to stratum corneum integrity, as detailed in a previous communication.^35^ Baseline resistance was measured before skin exposure and divided by measured resistance following exposure to ammonia.

### Statistical Analysis

Graphs were produced using GraphPad Prism v. 9.4.1 and statistical analysis was completed using GraphPad Prism. Methods of calculations are indicated in the text and a *p* ≤ 0.05 was considered to be statistically significant.

## Conclusions

This work has shown that in silico docking experiments predicted urease inhibitory action of compounds within *N. officinale* extract: ITCs and flavonoids. In vitro experiments demonstrated the extract’s ability to inhibit urease. Furthermore, ITCs molecules can sequester ammonia; as demonstrated, with PE-ITC via thioamide bond formation. In vivo human and ex-vivo porcine studies of *N. officinale* extract demonstrated potential anti-inflammatory properties on human skin, seen as reduction in erythema and a reduction in decrease in skin barrier resistance. *N. officinale* extract has interesting biological properties, with inhibitory activity against urease. It could be effective in treating or preventing various infections such as *H. pylori* gastric infection, CAUTI, and IAD. One weakness of the above approach is that at the time of writing it was not possible to dock the putative thiol-urea adducts, which may themselves have a significant interaction with urease.

## Supplementary Material



## Notes

Primary list of ligands identified to potentially be present in *N. officinale* *extract*, binding site and the docking score; alignment of the protein sequences of the alpha subunit of urease from different bacterial species (PDF)
