# Supplementary material for: Urease Inhibitors from Nasturtium officinale Extract for the Treatment of Urease Expressing Bacterial Infections
Source: ACS Pharmacol Transl Sci. 2026 Jun 14;9(7):1795–803. doi: 10.1021/acsptsci.5c00809 (PMC13366345; doi:10.1021/acsptsci.5c00809)
Supplement: Supplementary file 1 [file pt5c00809_si_001.pdf]

Supporting Information

Urease inhibitors from *Nasturtium officinale* extract for the treatment of urease expressing bacterial infections

Rachel Heylen[a], Emily Owen[a], Kyle Stewart[b], Paul G. Winyard[b], Andrew Tobias. A. Jenkins[a]*.

a. Department of Chemistry, University of Bath, Claverton Down, Bath, BA2 7AY, UK.
b. Watercress Research Ltd, Unit 24, Exeter SkyPark, Exeter, EX5 2FL, UK.

*Author to whom correspondence should be addressed: a.t.a.jenkins@bath.ac.uk

Table of Contents

Data	Page
Supplementary Table S1. List of ligands identified to potentially be present in *Nasturtium officinale* extract, the binding site and the docking score	S2
Supplementary Figure S1. An alignment of the protein sequences of the alpha subunit of urease from different bacterial species	S4
Supplementary Figure S2. Control compounds docked to urease: AHA and Urea	S5
Supplementary Figure S3. Cysteine residues identified on the surface of Helicobacter pylori urease	S6
Supplementary Figure S4. Quercetin docked into the active site of *Helicobacter pylori*	S7
Supplementary Figure S5. Minimum inhibitory concentration of *Nasturtium officinale* extract measured against *Proteus mirabilis*	S8
Supplementary Figure S6. H1-NMR Spectra demonstrating the formation of the thioamide bond	S10
Supplementary Figure S7. Macroscopic imaging of the ventral forearms of one individual after being exposed to solutions of artificial urine in the absence/presence of P. mirabilis, or artificial urine with *Proteus mirabilis* in the presence of *Nasturtium officinale*/acetohydroxamic acid.	S10
References	S11

Supplementary Table S1. **List of ligands identified to potentially be present in *Nasturtium officinale* extract, the binding site and the docking score**. Molecules were docked using Cresset Flare v. 4.0.2 software.

**Ligand**	**Docking score**	**Predicted targeted Binding site**
**Isothiocyanates** (Fahey et al., 2013; Klimek-Szczykutowicz et al., 2018; Panahi Kokhdan et al., 2021)		
3(methylsulfinyl)propyl-ITC	C153: -4.602<br>C257: -2.869<br>C321: -5.452	Covalent docking C153, C257, and C321
4(methylsulfinyl)butyl-ITC	C153: -4.876<br>C257: -3.429<br>C321: -5.874	Covalent docking C153, C257, and C321
5(methylsulfinyl)pentyl-ITC	C153: -5.561<br>C257: -3.619<br>C321: -6.164	Covalent docking C153, C257, and C321
6(methylsulfinyl)hexyl-ITC	C153: -5.683<br>C257: -5.615<br>C321: -6.194	Covalent docking C153, C257, and C321
7(methylsulfinyl)heptyl-ITC	C153: -5.955<br>C257: -3.702<br>C321: -6.822	Covalent docking C153, C257, and C321
8(methylsulfinyl)octyl-ITC	C153: -6.558<br>C257: -4.014<br>C321: -5.381	Covalent docking C153, C257, and C321
3(methylthio)propyl-ITC	C153: -2.167<br>C257: -3.196<br>C321: -5.773	Covalent docking C153, C257, and C321
4(methylthio)butyl-ITC	C153: -5.833<br>C257: -3.403<br>C321: -6.441	Covalent docking C153, C257, and C321
5(methylthio)pentyl-ITC	C153: -6.497<br>C257: -3.469<br>C321: -5.876	Covalent docking C153, C257, and C321
6(methylthio)hexyl-ITC	C153: -6.760<br>C257: -4.556<br>C321: -6.694	Covalent docking C153, C257, and C321
7(methylthio)heptyl-ITC	C153: -6.822<br>C257: -5.750<br>C321: -6.897	Covalent docking C153, C257, and C321
8(methylthio)octyl-ITC	C153: -7.006<br>C257: -5.952<br>C321: -6.078	Covalent docking C153, C257, and C321
(2-isothiocyanethyl)benzene	C153: -5.111<br>C257: -3.749<br>C321: -4.670	Covalent docking C153, C257, and C321

(3-isothiocyanatopropyl)benzene	C153: -4.862<br>C257: -3.450<br>C321: -4.423	Covalent docking C153, C257, and C321
(4-isothiocyanatobutyl)benzene	C153: -5.511<br>C257: -3.682<br>C321: -4.733	Covalent docking C153, C257, and C321
(5-isothiocyanatopentyl)benzene	C153: -5.862<br>C257: -3.499<br>C321: -4.814	Covalent docking C153, C257, and C321
(6-isothiocyanatohexyl)benzene	C153: -6.196<br>C257: -3.893<br>C321: -5.097	Covalent docking C153, C257, and C321
(7-isothiocyanatoheptyl)benzene	C153: -6.738<br>C257: -4.964<br>C321: -5.125	Covalent docking C153, C257, and C321
(8-isothiocyanatooctyl)benzene	C153: -6.831<br>C257: -4.624<br>C321: -5.858	Covalent docking C153, C257, and C321
**Flavonoids** (Boligon et al., 2013; Klimek-Szczykutowicz et al., 2018; Panahi Kokhdan et al., 2021)		
Quercetin	-8.299	Active site
Quercetin-3-O-sophoroside	-9.979	Active site and flap
Quercetin-3-O-sophoroside, 7-O-glucoside	-9.131	Active site and flap
Quercetin-3-O-Glc-(6′-malonyl-Glc)	-8.204	Active site
Quercetin-3-O-rutinoside (Rutin)	-9.559	Active site and flap
Kaempferol	-7.840	Active site
Luteolin	-8.972	Active site and flap
Syringetin	-9.060	Active site
Tricin	-8.517	Active site
Naringenin	-8.807	Active site
Hesperetin	-9.280	Active site and flap
Apigenin	-7.352	Active site

A range of biochemical components of interest was identified by UHPLC-QTOF mass spectrometry. These analytes were screened in positive and negative modes and tentatively identified using the extracted ion chromatogram (EIC) function. A detailed account of the extraction methodology and UHPLC-QToF analysis of watercress extract are provided in a separate publication [Winyard et al, 2025]. The principal active components found in aqueous watercress extract were: Isothiocyanates: PTCG (β-phenylethyl–glutathione); Phenethyl isothiocyanate (PEITC); 7-methylsulfinylheptyl isothiocyanate; 8-methylsulfinyloctyl isothiocyanate; Sulforaphane. Myrosinase activity, likely from myrosinase released on the crushing of plant leaves in watercress converts glucosinolates to isothiocyanates was detected. Glucosinolates: 1-Methoxyglucobrassicin; 4-Methoxyglucobrassicin; 4-Hydroxyglucobrassicin.

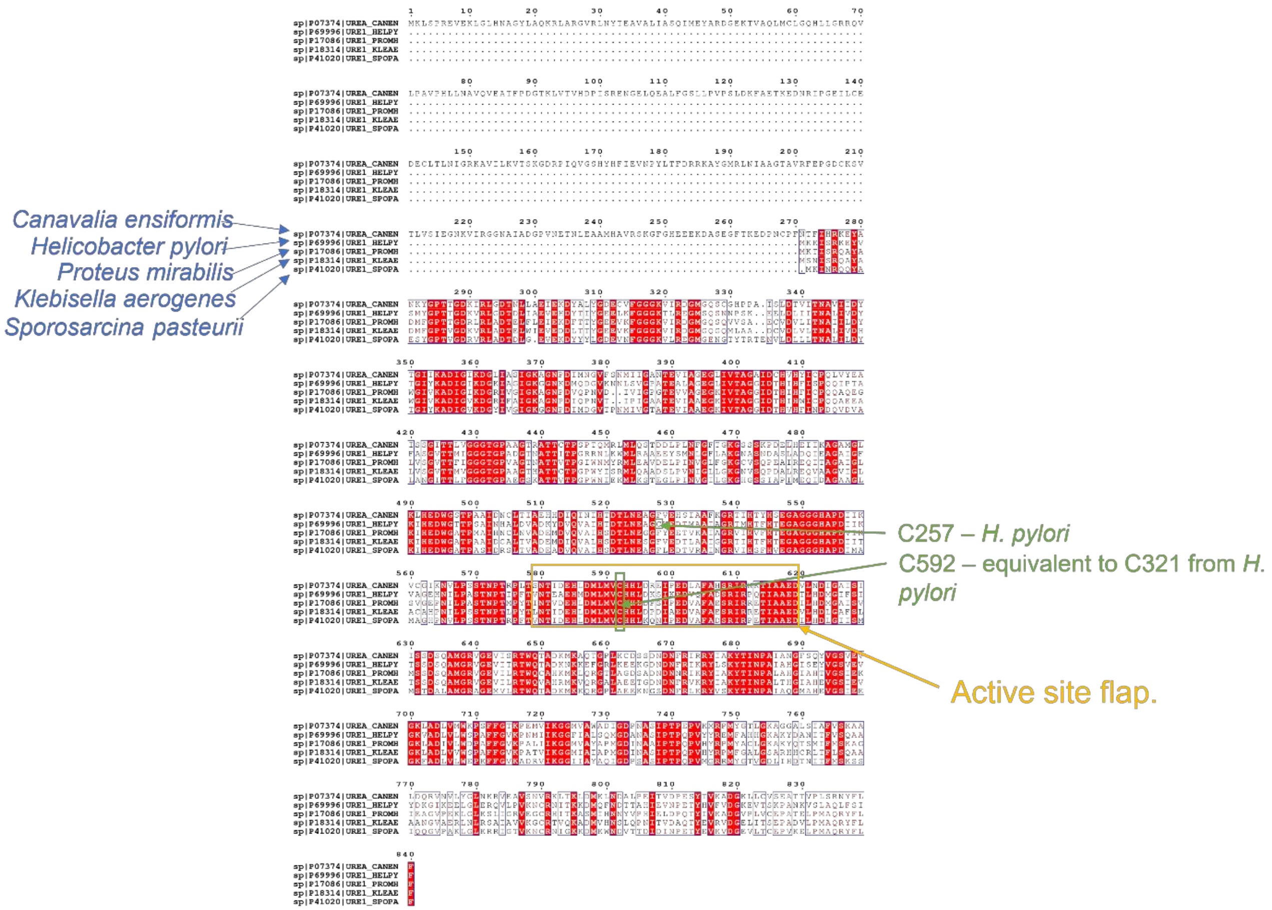

Supplementary Figure S1. An alignment of the protein sequences of the alpha subunit of urease from different bacterial species. P07374 is the sequence from *Canavalia ensiformis*; P69996 is from *Helicobacter pylori*; P17086 is from *Proteus mirabilis* (strain HI4320); P18314 is from *Klebsiella aerogenes*; and P41020 is from *Sporosarcina pasteurii* (formally known as *Bacillus pasterii*); and. Sequences were obtained from the Uniprot database.(Consortium, 2019) The green boxes identify cysteine residues which are found on the surface of the protein. The yellow box identifies amino acids involved in the formation of the active site flap. (Benini et al., 2000) The alignments were carried out using Multalin (Corpet, 1988) and presented using ENDscript. (Robert and Gouet, 2014)

A B

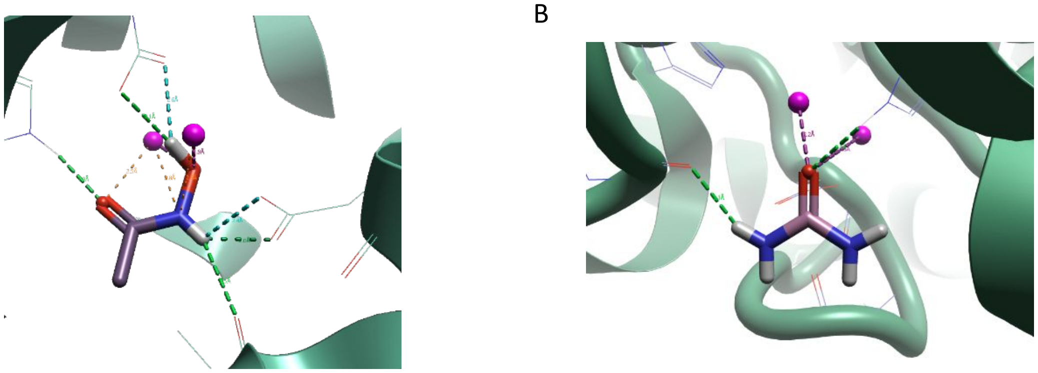

Supplementary Figure S2. Control compounds docked to urease: AHA and Urea. A. Acetohydroxamic acid bound to the active site of urease from *Helicobacter pylori* (PDB: 1E9Y). Coordinates with Ni^{2+} ion with bonds to A365 (2.5 Å), H221 (1.9 Å), K219 (2.19 Å), and D362 (2.0 Å), comparable to crystal structures of AHA bound to urease.(Ha et al., 2001) **B.** urea docked into the active site, chelates with the Ni^{2+} ions and coordinates with expected amino acids: A365 (2.1 Å), H221 (2.0 Å), H274 (2.4 Å), G279 (2.4 Å), and D362 (2.4 Å). Urease represented with green ribbon, Ni^{2+} ions as pink spheres, close contacts shown in thin line, docked ligands as thick lines. The molecules were docked by using Cresset Flare v. 4.0.2. The images were generated using Flare™ from Cresset®.

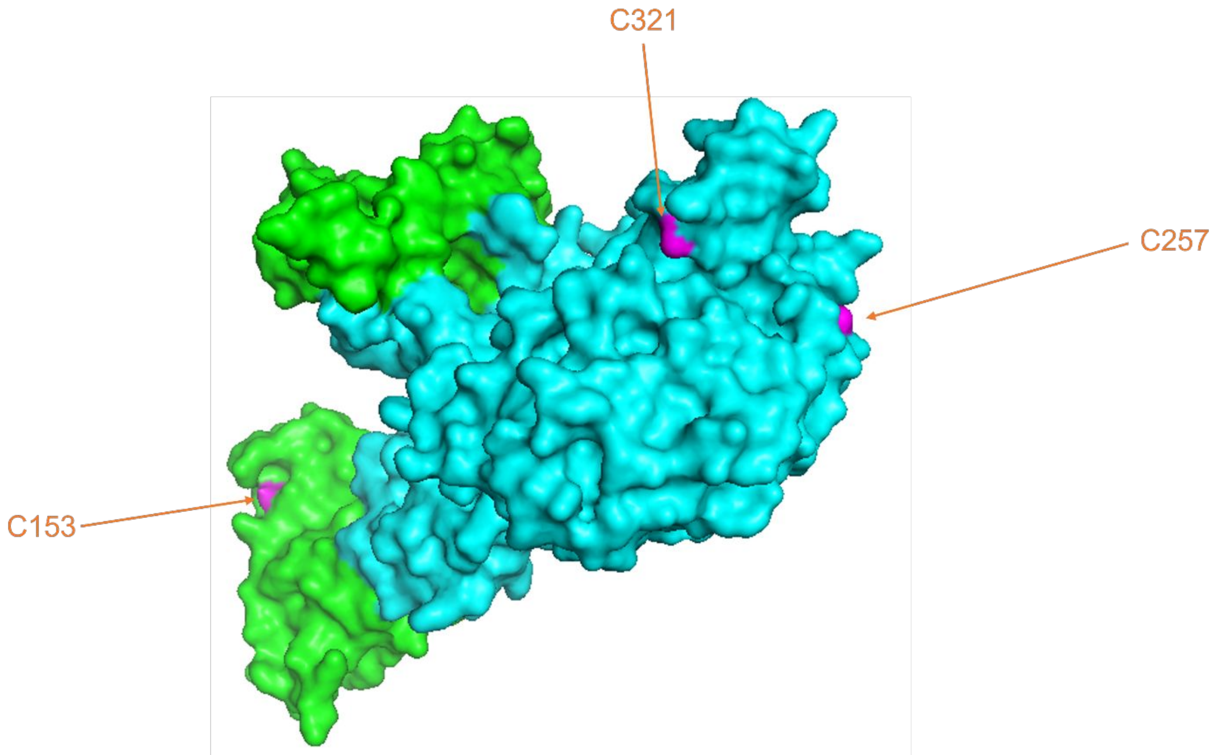

Supplementary Figure S3. Cysteine residues identified on the surface of *Helicobacter pylori* urease. Chain A in green, chain B in blue, and cysteine residues in magenta. The image was generated using Flare™ from Cresset®.

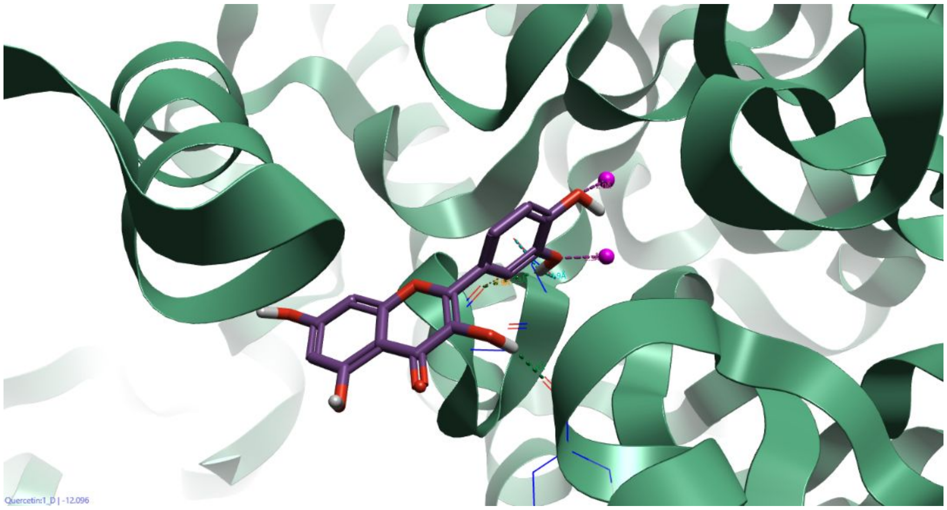

Supplementary Figure S4. Quercetin docked into the active site of *Helicobacter pylori* urease. Quercetin interacts with Ni^{2+} ions, D168, N362, and D365. The close contacts are shown as thin lines, whilst the docked ligands are shown as thick lines. The molecule was docked and the image generated by using Cresset Flare v. 4.0.2.

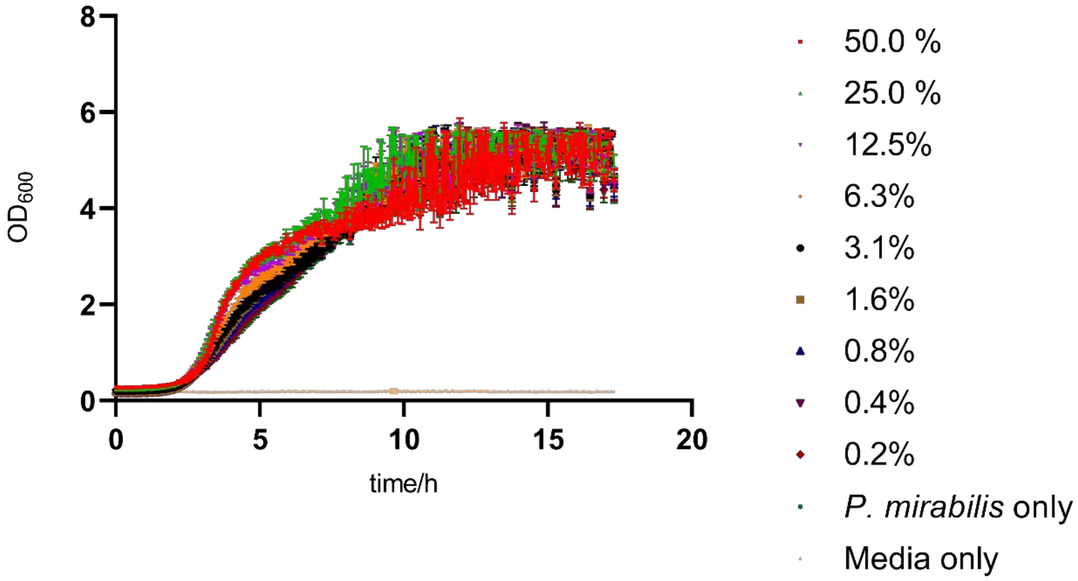

Supplementary Figure S5. Minimum inhibitory concentration of *Nasturtium officinale* extract measured against *Proteus mirabilis.* Experiments were completed as three biological repeats, each consisting of two technical repeats. The graphs show the mean of the biological repeats with error bars representing the standard deviations. No growth inhibition was observed at these concentrations up to 50%.

A.

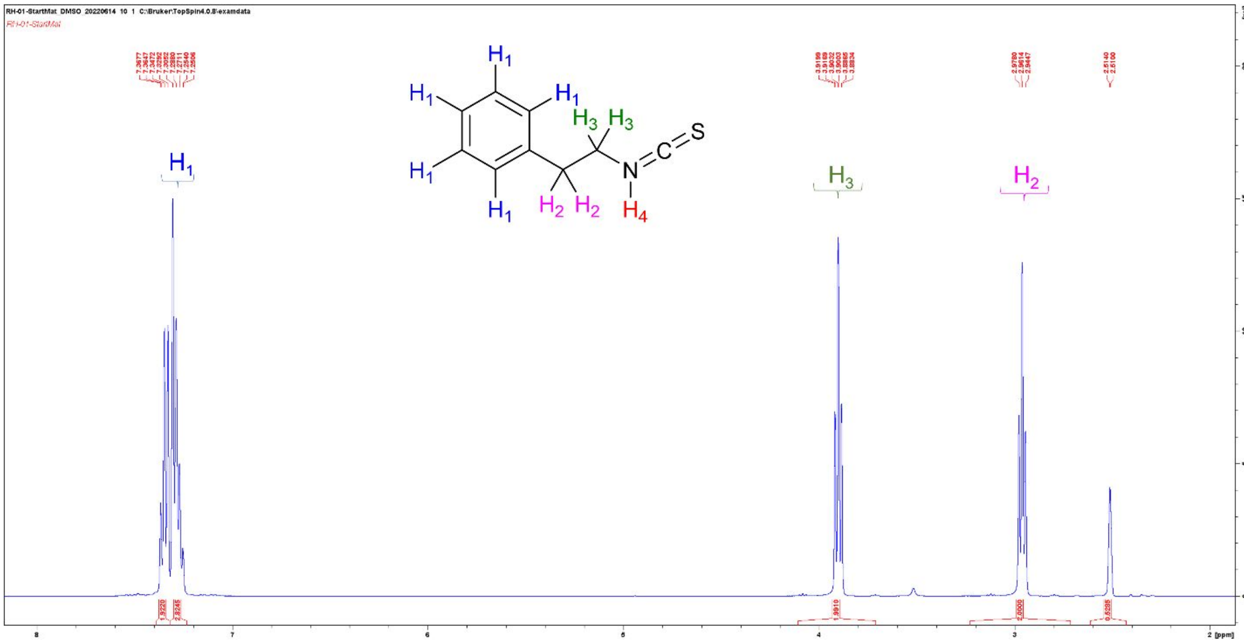
RH-01-StartMat_DMSO_20220614 10 1 C:\Bruker\TopSpin4.0.8\examdata
H1
H2
H3
H4
N=C=S
[ppm]
[rel]

B.

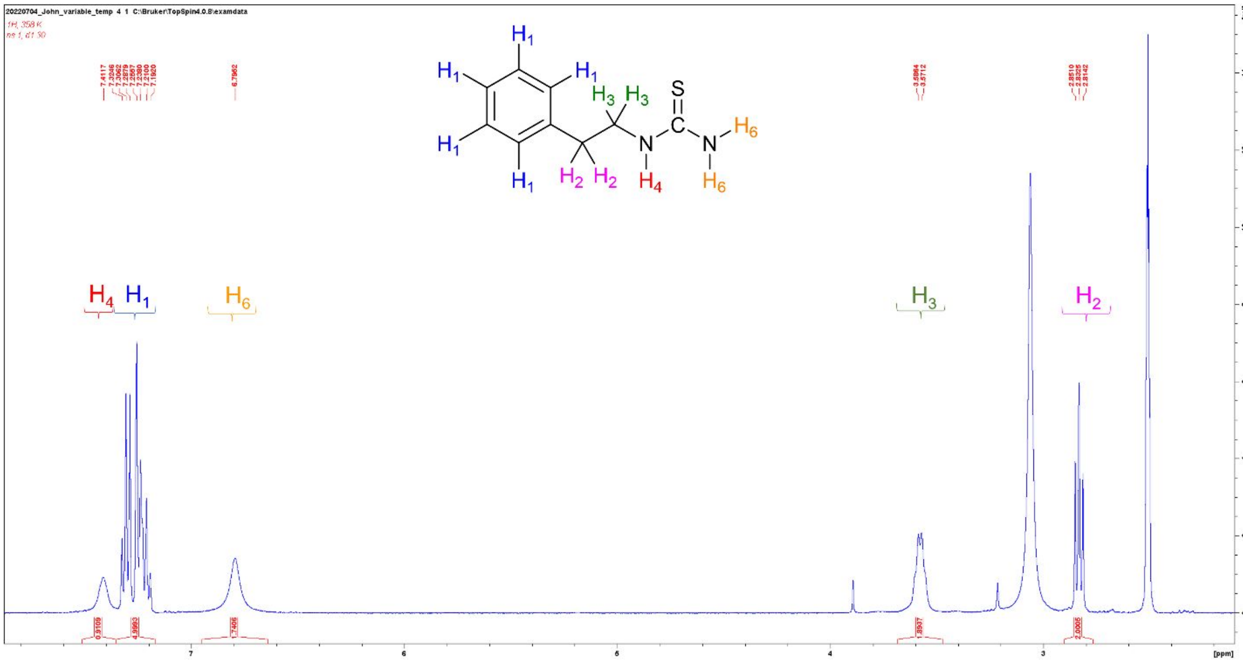
20220704_John_variable_temp 4 1 C:\Bruker\TopSpin4.0.8\examdata
H1
H2
H3
H4
H6
S
N
[ppm]
[rel]

C.

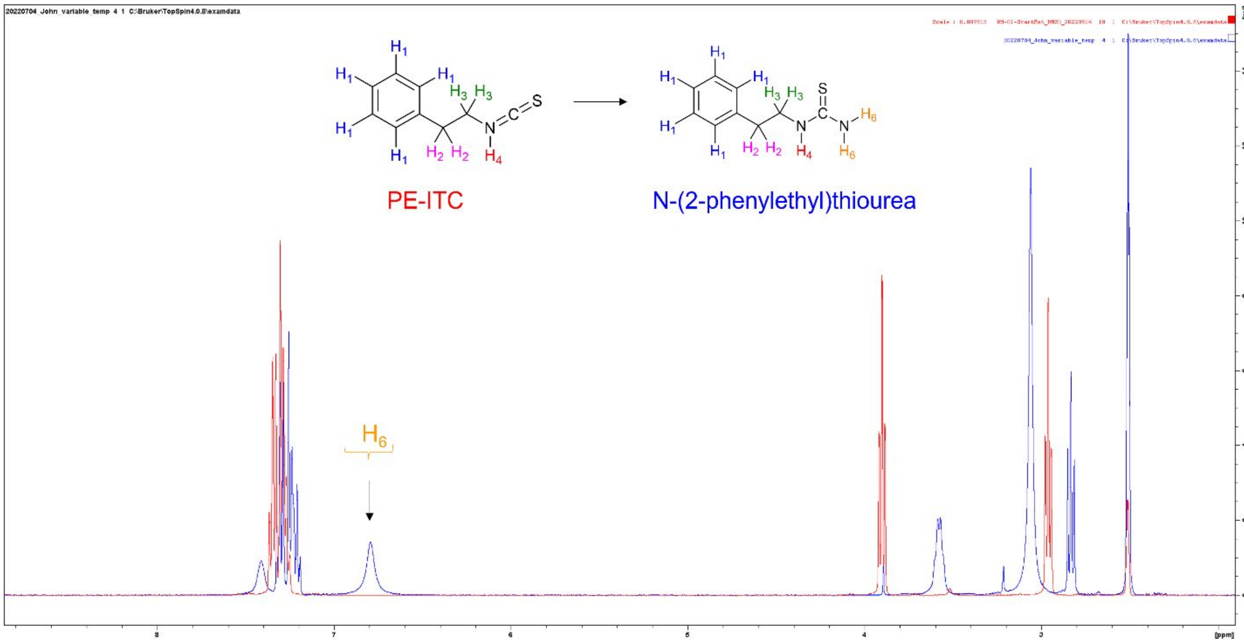

Supplementary Figure S6. H^1-NMR Spectra demonstrating the formation of the thioamide bond. A. Spectra of phenethyl-isothiocyanate (PE-ITC). **B.** Spectra of 1-phenethylthiourea. **C.** Overlay H^1-NMR spectra of PE-ITC (red) and 1-phenethylthiourea (blue). NMR spectra were acquired using a Bruker 500 MHz spectrometer in CD_3OD and processed by TopSpin 4.0.8.

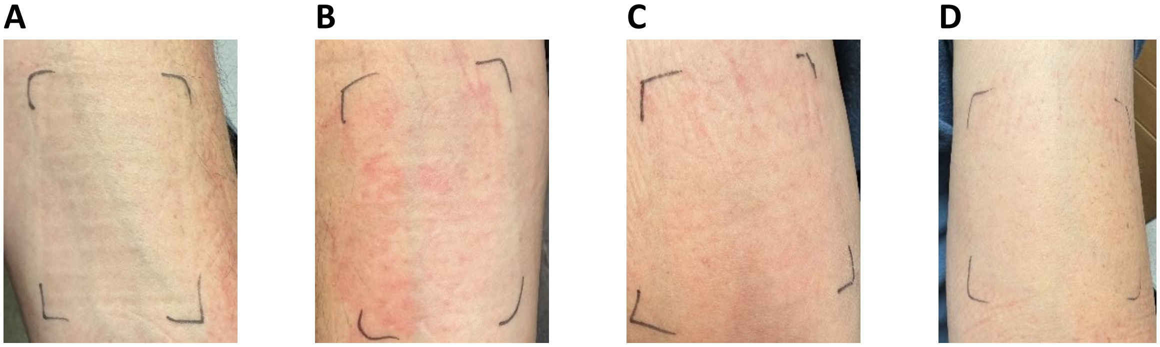

Supplementary Figure S7. Macroscopic imaging of the ventral forearms of one individual after being exposed to solutions of artificial urine in the absence/presence of *P. mirabilis*, or artificial urine with *Proteus mirabilis* in the presence of *Nasturtium officinale*/acetohydroxamic acid. A**.** artificial urine only. **B.** artificial urine with *P. mirabilis* B4 (10^9 CFU / mL). **C.** *P. mirabilis* and *N. officinale* (25% v/v). **D.** *P. mirabilis* and acetohydroxamic acid, AHA (5 mM) for 4 h.

References

Corpet, F., 1988. Multiple sequence alignment with hierarchical clustering. Nucleic Acids Res. 16, 10881–10890.

Robert, X., Gouet, P., 2014. Deciphering key features in protein structures with the new ENDscript server. Nucleic Acids Res. 42, 320–324. https://doi.org/10.1093/nar/gku316
